# Sirtuins and Cancer: Role in the Epithelial-Mesenchymal Transition

**DOI:** 10.1155/2016/3031459

**Published:** 2016-06-09

**Authors:** Raffaele Palmirotta, Mauro Cives, David Della-Morte, Barbara Capuani, Davide Lauro, Fiorella Guadagni, Franco Silvestris

**Affiliations:** ^1^Department of Biomedical Sciences and Human Oncology, University of Bari “Aldo Moro”, 70124 Bari, Italy; ^2^Department of Systems Medicine, School of Medicine, University of Rome Tor Vergata, 00133 Rome, Italy; ^3^IRCCS San Raffaele Pisana, 00166 Rome, Italy; ^4^University San Raffaele, 00166 Rome, Italy

## Abstract

The human sirtuins (SIRT1–SIRT7) enzymes are a highly conserved family of NAD^+^-dependent histone deacetylases, which play a critical role in the regulation of a large number of metabolic pathways involved in stress response and aging. Cancer is an age-associated disease, and sirtuins may have a considerable impact on a plethora of processes that regulate tumorigenesis. In particular, growing evidence suggests that sirtuins may modulate epithelial plasticity by inducing transcriptional reprogramming leading to epithelial-mesenchymal transition (EMT), invasion, and metastases. Though commonly regarded as EMT inducers, sirtuins may also suppress this process, and their functional properties seem to largely depend on the cellular context, stage of cancer development, tissue of origin, and microenvironment architecture. Here, we review the role of sirtuins in cancer biology with particular emphasis on their role in EMT.

## 1. Introduction: The Seven Sirtuins

The 7 mammalian sirtuins (Sirts) belong to a family of histone deacetylases (HDACs) that are ubiquitously expressed in different tissues and are classified as class I (Sirt1, Sirt2, and Sirt3), class II (Sirt4), class III (Sirt5), and class IV (Sirt6 and Sirt7) [1]. Sirtuins possess NAD^+^-dependent deacetylase activity and are implicated in many cellular processes such as cell cycle regulation, fatty acid metabolism, gene transcription, and cellular stress response [2].

Sirt1 is the most studied sirtuin protein, and its tissue expression is regulated by caloric restriction (CR) [3]. Sirt1 plays a pivotal role in regulating senescence and has been demonstrated to have an antiaging effect by reducing inflammation and oxidative stress [4]. The activation of Sirt1 by CR or resveratrol, a powerful natural activator of Sirt1, significantly increases the lifespan [5]. Among the possible mechanisms responsible for this beneficial effect, the significant reduction of reactive oxygen species (ROS) production and therefore the oxidative stress cellular damage plays a central role [6].

In mammals, Sirt1 seems to have a more complex role in control of metabolism. In *β*-cell-specific Sirt1-overexpressing (BESTO) transgenic mice, Sirt1 is able to increase insulin secretion in response to glucose [7, 8]. This response is accompanied by a decrease of the expression of uncoupling protein-2 (UCP-2), with consequent increased ATP production and cell survival. In the rat brain, activation of Sirt1 by low-dose resveratrol has been shown to mimic the beneficial effect of ischemic preconditioning and to protect the neurons after cerebral ischemia [9].

Sirt2 plays an important role in controlling cell cycle; in fact, an increase of Sirt2 activity significantly delays cell cycle progression [10, 11].

The mitochondrial Sirt3 deacetylase has been implicated in controlling longevity through decreasing ROS production as well as Sirt1 [12, 13]. Sirt3 deacetylates and activates mitochondrial enzymes involved in fatty acid *β*-oxidation, amino acid metabolism, electron transport chain, and antioxidant defenses [14]. Sirt3 has been related also to adaptative thermogenesis because of its regulation in both white and brown adipose tissue by CR and cold exposure [15]. Sirt3 is able to activate many cellular pathways by regulating mitochondrial genes such as PGC-1*α* and UCP-1 [14].

Sirt4 is a mitochondrial sirtuin lacking* in vitro* deacetylase activity [16]. It ADP-ribosylates and inhibits the mitochondrial glutamate dehydrogenase (GDH), thus regulating glutamine and glutamate oxidative metabolism and amino acid-stimulated insulin secretion [17]. The main target of Sirt5 in the mitochondria is the urea cycle enzyme carbamoyl phosphate synthetase 1 (CPS-1) [18]. By the activation of CPS-1, Sirt5 catalyzes ammonia to urea and reduces the production of oxidative stress having a cellular protective effect.

Sirt6 controls genomic DNA stability and repair [1]. Sirt6 was initially described as an exclusive ADP-ribosyltransferase [19], but recently its activity has been demonstrated as histone deacetylase [20]. By its effect on DNA repair, Sirt6 could play an essential role in maintaining organ integrity.

Sirt7 is the only sirtuin localized in the nucleolus [1] and is a component of the RNA polymerase I (Pol I) transcriptional machinery. By interacting with RNA Pol I and histones, Sirt7 regulates the transcription of rDNA in mammal cells [21].

Sirtuins have been associated with vascular diseases in humans. By using a multiethnic cohort from the Northern Manhattan Study (NOMAS), we demonstrated that genetic variants from the different sirtuins were significantly associated with the risk of phenotypes of atherosclerosis measured as carotid plaque [22], carotid intima media thickness [23], arterial stiffness [24], and plaque area and morphology [25]. Moreover, we showed a direct interaction between sirtuins and vascular risk factors such as hypertension and diabetes suggesting that these proteins have a fundamental role in developing or protecting from chronic diseases.

## 2. Sirtuins and Cancer

Mammalian Sirts regulate different and important cell functions, which may have an important role in cancer: chromatin regulation, cell survival, metabolic homeostasis, development, and cell differentiation [26]. Interestingly, Sirts seem to have a dual role in cancer. In fact, while protecting the organism against tumors by increasing genomic stability and limiting cellular replicative lifespan, they can also induce tumorigenesis by promoting cell survival under stress conditions and improving the uncontrolled cell division [27]. The possible explanation of this double face of Sirts in cancer could be related to their key role in cellular pathways such as cell growth, cell cycle, genome integrity, and cell death in response to stressor stimuli [20].

Sirt1 and the other Sirts have been shown to have both pro- and anticarcinogenic effects by improving genetic stability and regulating pathways that contribute to tumor suppression. Particularly, Sirt1 inhibits NF-*κ*B, which is a promoter of inflammation, survival, and cancer metastasis [28].

Similar to Sirt1, Sirt2 is either a positive or a negative regulator of the tumorigenic process. Sirt2 has a pivotal role in controlling the cell cycle. In particular, during mitosis, Sirt2 shuttles from the cytoplasm to the nucleus, where it binds chromatin [10] and deacetylates H4-K16, thus contributing to chromatin condensation during the G2/M transition [29]. When Sirt2 is overexpressed, the end of mitotic phases is delayed, hindering chromatin condensation and slowing the cell proliferation [10]. In fact, its low expression is observed in gliomas [30], breast cancer, and head and neck carcinomas with a coherent loss of Sirt2 enzymatic activity [31].

Sirt3 induces apoptosis or cell survival under normal or stress conditions, respectively [32]. Since its localization is mitochondrial, Sirt3 presents a tumor suppressor function mainly through mechanisms linked to oxidative response, energetic balance, and metabolic regulation [33, 34]. Decreased levels of Sirt3 enhance ROS production and lower the activity of relevant antioxidant enzymes such as superoxide dismutase 2 (SOD2), mitochondrial isocitrate dehydrogenase 2, and FOXO3a. This increase in ROS levels promotes genomic and mitochondrial DNA instability with consequent tumor progression [35, 36]. Recent findings clarify the protective effect of Sirt4 against cell death induced by genotoxic stress. In particular, it appears that Sirt4 preserves NAD^+^ levels through nicotinamide phosphoribosyltransferase (NAMPT) activity [37], while loss of Sirt4 enhances glutamine metabolism, leading to genomic instability and oncogenic phenotype [38], thereby suggesting a protective role of this protein against cancer. However, to date, there are no studies on the role of Sirt5 in cancer.

Differently from other Sirts, Sirt6 is a well-established tumor suppressor. Several studies conducted in different human tumors showed a decrease of Sirt6 expression in the pathological tissue, while the overexpression of this sirtuin increased the cell apoptosis levels [39]. Sirt6 has only deacetylase activity, binds HIF1 to promoter targets, and modulates glycolytic genes by H3K9 deacetylation [40].

The analysis of Sirt7 expression showed high levels of its mRNA in breast, thyroid, and hepatic cancers [41, 42], suggesting an implication of this protein in cell transformation. Sirt7 inhibits the cell cycle and promotes apoptosis, mainly through H3K18 deacetylation [43]. Similar to the other Sirts, Sirt7 has been considered a tumor suppressor and its activity is mediated by the negative regulation of HIF1 and HIF2 transcription [44].

## 3. EMT: A Central Regulator of Cancer Progression

The epithelial-mesenchymal transition (EMT) is a naturally occurring transdifferentiation program that governs changes of cell states along the epithelial versus mesenchymal axis and confers epithelial-mesenchymal plasticity to epithelial cells [45]. During EMT, epithelial cells lose their junctions and apical-basal polarity, reorganize their cytoskeleton, and undergo modifications in signaling, leading to widespread epigenetic reprogramming of gene expression. This, in turn, increases the motility of individual cells, enables the development of an invasive phenotype, induces resistance to senescence and apoptosis, and confers immunosuppressive capabilities, pluripotency, and stem-like properties [46].

The transition of epithelial cells into mesenchymal-like cells follows a highly conserved program and is a crucial event of the invasion-metastasis cascade [47]. However, the depiction of EMT as a binary switch that moves cells from a fully epithelial to a fully mesenchymal state does not reflect the actual mechanisms underlying this process. In fact, depending on the tissue and the signaling context, epithelial cells may lose only some characteristics, thus showing both epithelial and mesenchymal features concomitantly (partial EMT). Nonetheless, the acquisition of even a subset of mesenchymal traits endows cells previously residing in a fully epithelial state with a suite of features that have profound implications on their biology [45, 48].

The EMT program can be activated with remarkable rapidity in epithelial cells. Such rapid interconversion between epithelial and mesenchymal states implies plasticity in response to EMT-inducing signals, thus suggesting that residence in these states is metastable and governed by transient and complex cellular and molecular mechanisms [45]. A plethora of heterotypic signals is able to induce EMT in cancer cells, including transforming growth factor-*β*1 (TGF-*β*1), epidermal growth factor (EGF), fibroblast growth factor (FGF), hepatocyte growth factor (HGF), insulin-like growth factor-1 (IGF-1), vascular endothelial growth factor (VEGF), and platelet-derived growth factor (PDGF), as well as prostaglandin E2 (PGE2), cytokines, and morphogens such as Wnt, Notch, and Sonic hedgehog (Shh). Mechanistically, the activation of EMT is orchestrated by a network of EMT-related transcription factors (EMT-TFs) that interact with epigenetic regulators to control the expression of proteins involved in cell polarity, cell-cell contact, cytoskeleton architecture, and extracellular matrix degradation [49]. Since the loss of E-cadherin expression is considered a crucial event in EMT, EMT-TFs have been classified based on their ability to repress* E-cadherin* directly (SNAIL, SLUG, ZEB1, ZEB2, etc.) or indirectly (TWIST1, TWIST2, E2.2 SIX1, FOXC2, etc.) [46]. In addition to the direct effects of EMT-TFs on gene expression, changes at the RNA levels can regulate EMT. In particular, noncoding miRNAs can selectively bind and inhibit the translation of mRNAs of EMT master transcription factors and/or of genes defining the epithelial phenotype (including those encoding adhesion junctions, polarity complex proteins, and signaling mediators). Moreover, differential splicing of nascent RNAs operates during EMT and can drive an extensive switch between epithelial- and mesenchymal-specific protein isoforms [46, 50, 51]. Beyond the mRNA level, modifications in chromatin configuration have been described as an essential determinant for the long-term residency of cells in a given phenotypic state during EMT, as the gain of an increasingly stable mesenchymal phenotype largely relies on different rounds of histone acetylation and DNA methylation, driving the interconversion of facultative heterochromatin into an active euchromatic state and vice versa [49, 52]. In this multifaceted context, accumulating evidence points to sirtuins as key epigenetic modulators of EMT activation and maintenance.

## 4. Sirtuins and EMT

**Figure 1:**
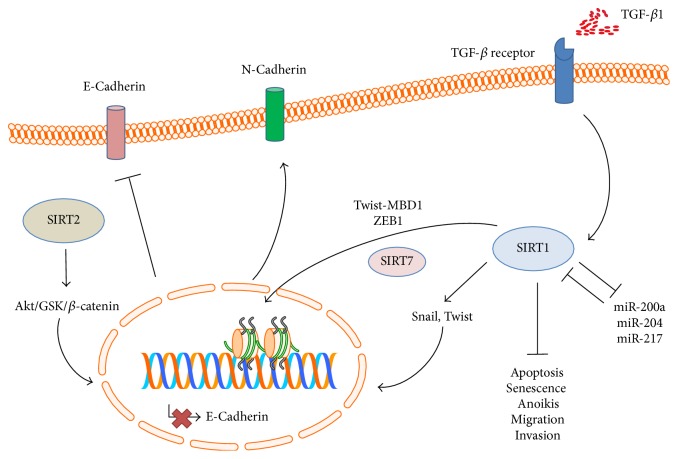
Positive regulation of EMT by sirtuins: selected pathways. Activation of TGF-*β* signaling upregulates SIRT1 expression, thus increasing cellular resistance to apoptosis, senescence, and anoikis and reducing migration and invasion capabilities. By interacting with SIRT7, Zeb1, and the Twist-MBD1 complex, SIRT1 represses the transcription of epithelial genes including* E-cadherin*, while increasing the expression of mesenchymal genes such as N-cadherin and Vimentin. SIRT1 can also upregulate the EMT master regulators Snail and Twist, further suppressing E-cadherin expression. Several miRNAs, including miR-200a, miR-204, and miR-217, have been described as negative regulators of SIRT1. SIRT2 can activate the Akt/GSK/*β*-catenin signaling, thus positively regulating EMT and metastatic potential. Different pathways may be active in different cancers; refer to the text for further details.

**Figure 2:**
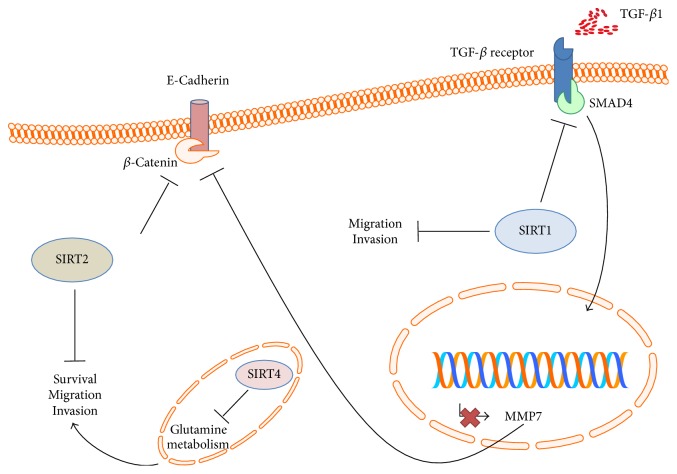
Negative modulation of EMT by sirtuins. SIRT1 can inhibit the TGF-*β* signaling pathway by deacetylating Smad4. This leads to decreased MMP7 transcription and expression, with consequent reduced E-cadherin degradation. Being bound to E-cadherin, *β*-catenin cannot shuttle to the nucleus, with subsequent lack of its prosurvival, EMT-inducing effects. Similarly, SIRT2 downregulates *β*-catenin, while promoting the expression of E-cadherin. By inhibiting the mitochondrial glutamine metabolism, SIRT4 suppresses EMT.

Sirtuins play complex roles in either promoting or suppressing EMT, and their functional properties may largely depend on the cellular context, stage of cancer development, tissue of origin, and microenvironment architecture. The signaling pathways evoked by sirtuins to activate or inhibit EMT in cancer cells are summarized in Figures 1 and 2, respectively.

### 4.1. Sirtuins as Inducers of EMT

The involvement of SIRT1 in EMT activation has been extensively studied during the past decade, and controversial results have been reported so far.

SIRT1 has been shown to regulate acinar-to-ductal metaplasia and promote tumorigenesis and metastases in pancreatic cancer [53, 54]. In particular, the TGF-*β*1 driven EMT of pancreatic cancer cells upregulates SIRT1 expression, while knockdown of the histone deacetylase is able to revert the cell phenotype via mesenchymal-epithelial transition (MET). Interestingly, miR-217 negatively regulates SIRT1 mRNA translation, thus suggesting dysregulation of the miR-217-SIRT1 axis in response to the inflammatory environment of pancreatic carcinoma [55]. At both mRNA and protein levels, SIRT1 overexpression in pancreatic cancer tissue is apparently associated with tumor size, stage, and presence of lymph node or liver metastases. Downregulation of SIRT1 by small hairpin (sh)-RNA increased E-cadherin expression while reducing tumor cell proliferation, invasion, metalloproteinase (MMP) expression, and capacity to form tumors* in vivo*, thus emphasizing the active role of the histone deacetylase in EMT induction [56]. Mechanistically,* E-cadherin* transcriptional repression has been directly related to SIRT1 in pancreatic cancer. In fact, by interacting with Twist and methyl-CpG binding domain protein-1 (MBD1), SIRT1 can form a protein complex capable of silencing the promoter of* E-cadherin* [57]. Pharmacological inhibition of Aurora kinase A (AURKA), a key cell cycle regulator critical for mitotic events, has been recently shown to suppress, at least partially, EMT in pancreatic as well as ovarian cancer cell lines. The effect has been ascribed to the observed modulation of the SIRT1-mediated pathway, but the signaling operating between AURKA and SIRT1 still remains elusive [58, 59]. Notably, treatment with the SIRT1 inhibitor EX527 reduced the proliferation of pancreatic cancer cells and enhanced their sensitivity to gemcitabine* in vitro*, but no apparent effects on EMT were seen. On the contrary,* in vivo* SIRT1 inhibition promoted xenograft tumor growth, thus suggesting possible off-target effects on the tumor microenvironment and adding another layer of complexity to the characterization of the EMT-related activity of sirtuins [60].

In hepatocellular carcinoma (HCC), SIRT1 is overexpressed in malignant tissue as compared with normal liver, and its expression is significantly correlated with tumor size, tumor number, stage, and poor prognosis [61]. The oncogenic activity of SIRT1 in HCC has been related to the induction of genomic instability via dysregulated telomeric maintenance [62] as well as EMT initiation via Snail and Twist upregulation, with consequent E-cadherin suppression. Consistently, SIRT1 was shown to induce resistance to senescence or apoptosis [62], promote cell migration and invasion, and affect the metastatic potential of HCC cells in an ectopic model of liver cancer metastasis [61].

Overexpression of SIRT1 has been detected in both gastric and gastroesophageal junction cancers and has been related to tumor stage and occurrence of lymph node metastases [63, 64]. Consistent with its EMT-inducing activity, SIRT1 was found to downregulate E-cadherin while increasing Vimentin expression. Moreover, the histone deacetylase positively regulated the migration and invasion of gastric cancer cells and induced resistance to anoikis. Of interest, miR-204, a member of the miR-200 family capable of targeting the 3′UTR of SIRT1, is commonly downregulated in gastric cancer and modulates the metastatic process by primarily interfering with the SIRT1-LKB1 axis [64]. Similar results have been also reported in osteosarcoma [65], although the possible occurrence of EMT in mesenchymal cells is still a matter of debate.

In breast cancer, a negative feedback loop exists between SIRT1 and miR-200a, an epithelial phenotype-defining miRNA. Knockdown of SIRT1 or restoration of miR-200a prevented the EMT-like transformation driven by TGF-*β* in normal mammary epithelial cells, as evidenced by decreased anchorage-independent growth and decreased cell migration. Consistently, miR-200a levels in tumor samples or blood were inversely associated with SIRT1 expression in patients with* in situ* or invasive breast carcinoma [66].

In prostate cancer, SIRT1 represses the epithelial morphology through its deacetylase activity. In fact, while cells transfected with wild-type SIRT1 were characterized by loose cell-cell contact and spindle-shaped morphology reminiscent of EMT, those transfected with a catalytically inactive SIRT1 failed to show any relevant changes. E-Cadherin, N-cadherin, fibronectin, and *γ*-catenin were major targets of SIRT1 during the regulation of EMT in prostate cancer cell lines, and the suppression of E-cadherin expression was related to direct modulation of the proximal promoter in an E-box dependent manner. Based on the results of coimmunoprecipitation and chromatin immunoprecipitation studies, SIRT1 was also shown to physically interact with the zinc finger transcription factor ZEB1, thus suggesting that transcriptional repression of E-cadherin might be caused by deacetylation of histone H3 at the gene promoter, with consequent reduced RNA polymerase binding [67]. In this context, SIRT1 has been also reported to induce aberrant, long-term, heritable silencing of* E-cadherin* after double-strand break of its promoter. In fact, permanent histone hypomethylation and recruitment of DNA methyltransferase-1 (DNMT-1) and DNMT-3B, with consequent chromatin condensation, have been described in a minority (~1%) of cells transfected with a damaged, exogenous promoter construct of* E-cadherin* [68].

Though less extensively investigated, other sirtuins are apparently involved in the EMT process. In particular, SIRT2 is upregulated in HCC, and its overexpression is associated with vascular invasion, advanced tumor stage, and shorter survival. The tumorigenic activity of SIRT2 in HCC has been related to EMT induction by direct targeting of the protein kinase B/glycogen synthase kinase (Akt/GSK)3-*β*/*β*-catenin signaling pathway [69]. Moreover, in colon cancer cells, pharmacological inhibition of SIRT2 by the benzylsulfonamide AK-1 is able to induce proteosomal degradation of Snail with consequent cell cycle arrest and impaired wound-healing activity, thus further suggesting a possible role of this sirtuin in EMT promotion [70]. SIRT7 has metastasis-promoting effects, upregulating the motility and invasiveness of cancer cells of either epithelial or mesenchymal origin* in vitro* and increasing their metastatic potential* in vivo*. This effect has been related to direct EMT induction by SIRT7, in cooperation with SIRT1. In particular, it has been proposed that SIRT1 might function as a scaffold protein recruiting SIRT7 to the* E-cadherin* promoter, where it mediates the deacetylation of histone H3K18Ac, with consequent transcriptional repression of downstream targets. Interestingly, SIRT7 expression was found to be higher in tumor samples rather than in normal tissue, and its levels were dramatically elevated in metastatic tissue as compared with primary tumors. Consistently, amplification of the SIRT7 gene was reported to occur exclusively in tumors that were metastatic and associated with poor survival [71]. In colorectal cancer, SIRT7 expression levels were significantly correlated with tumor stage, lymph node metastasis, and poor outcomes. Enhanced invasive phenotype, colony formation potential, and a shift from epithelial to mesenchymal markers were also observed in SIRT7-overexpressing cells [72].

### 4.2. Sirtuins as Repressors of EMT

According to their ambivalent role in oncoproteins or tumor suppressors depending on the cellular context and redox state, sirtuins have been also described as repressors of EMT. Elegant experiments by Simic et al. showed that reduced SIRT1 levels in breast cancer cells have a prometastatic effect in nude mice, while the loss of this histone deacetylase exacerbated mesenchymal deposition in a murine model of injury-induced kidney fibrosis. Repression of EMT in cancer and fibrosis was regarded as the result of SIRT1-mediated inhibition of the TGF-*β* signaling pathway via Smad4 deacetylation, with consequent decreased MMP7 expression, E-cadherin degradation, and *β*-catenin nuclear translocation [73].

Similar findings were also reported in oral squamous cell carcinoma, in which TGF-*β* signaling inhibition in cells overexpressing SIRT1 significantly suppressed migration and invasion abilities while restoring an epithelial phenotype [74]. In a more recent paper, Xiao et al. demonstrated that resveratrol (RSV), a stilbene polyphenol from wine and grapes, inhibits EMT in renal injury and fibrosis induced by the pathway TGF-*β*/Smad4/MMP7 by activating SIRT1 [75].

In both lung and ovarian cancer, SIRT1 represses EMT and antagonizes migration* in vitro* and metastases* in vivo*. While resveratrol, as sirtuin activator, attenuated metastasis formation by blocking EMT, hypoxia was found to inhibit SIRT1, thus favoring EMT. In particular, hypoxic stress was shown to downregulate the expression of SIRT1 by reducing the occupancy of the transcriptional activator Sp1 on the proximal promoter of the gene in a SUMOylation-dependent manner [76, 77].

More recently, it has also been shown that SIRT1 attenuates nasal polypogenesis, both in murine transgenic models with Sirt1 overexpression and in wild-type (WT) mice treated with resveratrol, by inhibition of hypoxia-inducible factor 1- (HIF-1-) induced EMT [78].

In nontransformed cells, SIRT2 deacetylates *β*-catenin, thus repressing its prosurvival downstream transcriptional program. Moreover, the histone deacetylase positively regulates E-cadherin, while reducing MMP9 expression as well as cellular migration and invasion [79]. The mitochondrial deacetylases SIRT3 and SIRT4 have been shown to contrast the EMT activation through metabolic reprogramming. In particular, SIRT3 opposes Warburg phenotype of both cancer and stromal cells and is downregulated in EMT-inducing cancer-associated fibroblasts (CAFs). The consequent shift toward more glycolytic metabolism allows CAFs to produce lactate, which shuttles back to cancer cells, fueling their proliferation [80]. On the other hand, SIRT4 has been shown to upregulate E-cadherin expression and suppress proliferation, migration, and invasion of colorectal cancer cell lines through inhibition of the glutamine metabolism. Accordingly, SIRT4 expression decreased with the progression of colon cancer, and its loss was reported to be predictive of poor outcome [81].

## 5. Conclusions

Studies of sirtuins are rapidly growing in the field of cancer and other diseases, but the exact role of sirtuins in the EMT process is still largely debated. SIRT1 and SIRT2 have shown both EMT-promoting and EMT-suppressing effects. On the other hand, SIRT7 has been related to EMT induction only, while SIRT3 and SIRT4 have been described as pure EMT repressors. Such dual role in tumorigenesis and EMT regulation is not unprecedented. For example, TGF-*β* has antiproliferative functions in normal cells and early-stage malignancies but becomes a potent EMT inducer at later stages of tumorigenesis [82]. Thus, the precise role of sirtuins in cancer may depend on the cellular and molecular context. Future studies should evaluate which physiological or pathological circumstances are able to influence the function of sirtuins in terms of EMT regulation.

Attempts to therapeutically target EMT as cancer hallmark have been quite disappointing so far, probably as a consequence of the multifaceted and transient nature of this process. Both activators and inhibitors of sirtuins have been developed or are under development and are claimed for possible use as EMT regulators. However, current sirtuin modulators are generally lacking in specificity and potency for human use [83], and more selective agents are needed before testing of their anti-EMT function will be feasible.

Innovative genetically engineered cell lines and animal models are needed to expand our knowledge on the EMT-related pathways modulated by sirtuins and to test whether a specific sirtuin mutational landscape is associated with an increased risk of cancer and/or metastasis through EMT promotion. In coming years, we expect to see a rapid growth in sirtuins research. There is still a long way to go before fully elucidating their role in EMT and cancer.
